# Dual‐task difficulties as a risk factor for unemployment in people with multiple sclerosis

**DOI:** 10.1002/brb3.3299

**Published:** 2023-10-25

**Authors:** Turhan Kahraman, Hasretgul Temiz, Zuhal Abasiyanik, Cavid Baba, Serkan Ozakbas

**Affiliations:** ^1^ Department of Physiotherapy and Rehabilitation, Faculty of Health Sciences Izmir Katip Celebi University Izmir Turkey; ^2^ Department of Health Professions, Faculty of Health and Education Manchester Metropolitan University Manchester UK; ^3^ Graduate School of Health Sciences Izmir Katip Celebi University Izmir Turkey; ^4^ REVAL Rehabilitation Research Center, Faculty of Rehabilitation Sciences Hasselt University Hasselt Belgium; ^5^ Graduate School of Health Sciences Dokuz Eylül University Izmir Turkey; ^6^ Department of Neurology, Faculty of Medicine Izmir University of Economics Izmir Turkey

**Keywords:** cognition, difficulty, dual‐task, motor, work

## Abstract

**Background:**

No study has investigated the impact of dual‐tasking difficulties as a risk factor for unemployment in people with multiple sclerosis (pwMS). The aim was to examine the influence of dual‐task performance on employment status and work difficulties and to identify the predictors of employment status in pwMS.

**Methods:**

Eighty‐four pwMS, including 42 employed and 42 unemployed, participated in the study. Dual‐task difficulties were assessed using the Dual‐task Impact on Daily‐living Activities‐Questionnaire (DIDA‐Q), while dual‐task performance was evaluated through the 30‐second Walk Test and Nine‐Hole Peg Test, incorporating a cognitive task. Walking and cognitive function were also measured.

**Results:**

Employed pwMS had better scores in walking, cognitive function, single and dual‐task performance than unemployed pwMS (*p* < .05). Lower scores in walking (odds ratio [OR] = 1.81, *p* < .001) and upper extremity‐related (OR = 1.44, *p* = .019) dual‐task performance and higher scores in the cognitive subscale of the DIDA‐Q questionnaire (OR = 1.20, *p* = .037) were significantly associated with higher odds of being unemployed. Among employed pwMS, DIDA‐Q subscales showed moderate‐to‐strong correlations with MSWSDQ‐23 scores. The other variables showed weak‐to‐moderate correlations with subscale and total scores of MSWSDQ‐23.

**Conclusion:**

Cognitive function, as opposed to motor function, has been found to be a significant predictor of unemployment in pwMS.

## 1 INTRODUCTION

Multiple sclerosis (MS) is a chronic, debilitating disease that affects the central nervous system, causing a range of physical and cognitive symptoms. MS is usually diagnosed during early adulthood, which can have an impact on work‐related activities and occupational outcomes (Bruno Kusznir et al., 2022). Several studies have investigated the impact of MS on work, finding that people with MS experience a high level of work‐related challenges such as reduced work productivity associated costs, presenteeism, absenteeism, and unpaid work loss (Conradsson et al., 2020; Ernstsson et al., 2016; Rodriguez Llorian et al., 2022; Vitturi et al., 2022).

These challenges can be attributed to a range of factors including physical, cognitive, and emotional symptoms. These factors include being female, having a low education level, experiencing high levels of neurological impairment, and having a progressive form of the disease (Benedict et al., 2005; Kornblith et al., 1986; Larocca et al., 1985). In addition, MS symptoms, such as walking difficulties, bladder/bowel incontinence, heat sensitivity, fatigue, and cognitive impairment, particularly with regards to processing speed and memory, also contribute to unemployment (Benedict et al., 2005; Julian et al., 2008; Morrow et al., 2010; O'Connor et al., 2005; Rodriguez Llorian et al., 2022; Simmons et al., 2010; Smith & Arnett, 2005; Strober et al., 2012). One aspect of cognitive impairment that has received increasing attention in the MS literature is dual tasking, which refers to the ability to perform two tasks simultaneously (Leone et al., 2015; Wajda & Sosnoff, 2015). Dual tasking is an essential aspect of daily life and work, as it allows individuals to carry out multiple tasks simultaneously, such as listening to a colleague while typing an email, driving, operating machinery, and participating in meetings or conversations while completing other tasks. However, in MS, dual tasking can be challenging, with studies showing that people with MS perform more poorly on dual‐tasking tests than healthy controls (Allali et al., 2014; Chamard Witkowski et al., 2019; Downer et al., 2016; Hamilton et al., 2009; Kirkland et al., 2015; Postigo‐Alonso et al., 2018; Wallin et al., 2022).

Understanding the relationship between work difficulties and dual tasking in MS is crucial for the development of effective rehabilitation and vocational interventions. Additionally, it may help people with MS and their employers develop strategies to optimize work performance. According to the best of our knowledge, no study investigated the dual‐task difficulties as a risk factor for unemployment in people with MS. Therefore, the aim was to examine the influence of dual‐task performance on employment status and work difficulties and to identify the predictors of employment status in people with MS.

## 2 MATERIALS & METHODS

### 2.1 Participants

This cross‐sectional study was conducted between January 2022 and August 2022. The study population comprised people who were diagnosed with MS by a specialist neurologist and were being routinely monitored in the MS Unit of the Neurology Department at Dokuz Eylül University Hospital.

Notably, there is a lack of previous research on the impact of dual‐task performance on employment status in people with MS. Nonetheless, Strober et al. (2012) previously conducted a study comparing employed and unemployed people with MS in terms of information processing speed assessed by the Symbol Digit Modalities Test (SDMT) and found that unemployed people had significantly lower processing speed (effect size = 0.80). While information processing speed is a variable linked to dual‐task performance, it does not measure dual‐task performance directly. Therefore, using G*Power version 3.1.9.7 (Dusseldorf University, Dusseldorf, Germany), a sample size of 84 people, with 42 people in each group, was calculated, assuming a power of 95%, a 0.05 error probability, and an effect size of 0.80.

The study included a total of 84 people, comprising 42 employed and 42 unemployed people with MS, who met the inclusion criteria. The inclusion criteria for the employed MS group were a diagnosis of MS according to the McDonald 2017 criteria (Thompson et al., 2018), the ability to read and understand Turkish, no relapse within the last 3 months, and having been employed for at least 2 years. In addition to these criteria, the inclusion criteria for the unemployed MS group were having worked for at least 2 years and having been unemployed for at least 6 months. The exclusion criteria were having a neurological disease other than MS, being diagnosed with severe cognitive or psychiatric disorders, being retired, or being a student.

This study received approval from the Noninvasive Research Ethics Board of Dokuz Eylül University with decision number 2021/35‐09 on December 01, 2021. The research was conducted in accordance with the ethical standards of the 1964 Helsinki Declaration (revised in Brazil in 2013), and informed consent was obtained from the participants.

### 2.2 Assessments

#### 2.2.1 Neurological disability

The Expanded Disability Status Scale (EDSS) is a scale that evaluates disease‐specific functional disability in MS (Kurtzke, 1983). The neurologist applied the EDSS, and the scoring was performed through a neurological examination of pyramidal, visual, sensory, cerebral, brainstem, cerebellar, bladder, and bowel functions, as well as an ambulation evaluation. The degree of disability is determined by a score ranging from 0 (normal) to 10 (death due to MS).

#### 2.2.2 Self‐reported dual‐task difficulties

The Dual‐task Impact on Daily‐living Activities‐Questionnaire (DIDA‐Q) is a questionnaire consisting of 19 questions about daily activities frequently performed in daily life. It is a valid and reliable self‐reported measure developed for the MS population (Pedullà et al., 2020). Patients were asked to rate how difficult each task is, ranging from “Not difficult at all (0)” to “Extremely difficult (4)” while performing each task. It has subscores for balance mobility, upper extremity, and cognitive domains. High scores indicate high levels of difficulty. The Turkish translation of the questionnaire has been conducted, and its psychometric properties were found good (Abasıyanık et al., 2023).

#### 2.2.3 Dual‐task performance during walking and upper extremity task

To evaluate the dual‐task performance during walking and upper extremity tasks, the 30‐second Walk Test (30s WT) and Nine‐Hole Peg Test (NHPT) were utilized, respectively. The 30s WT involved instructing the participants to walk as quickly as possible for 30 s along an unobstructed, 20‐m straight path, with the distance covered being recorded in meters. The secondary task involved a verbal word generation task, which was reported to be sensitive in evaluating dual‐task performance during the 30sWT. The participants were asked to generate as many words as possible, beginning with a given letter (“A”) during the 30s WT, with the number of correctly generated words and the walking distance being recorded (Postigo‐Alonso et al., 2018).

The NHPT is a validated and reliable test of manual dexterity in MS, consisting of a block with nine standard holes and nine standard pegs to be placed in those holes (Fischer et al., 1999). The test was administered in accordance with the standard protocol, with the time taken to complete the test being recorded in seconds using a stopwatch (Fischer et al., 1999). Two trials were performed for each extremity, starting with the dominant side, and the average was recorded as the score.

For the dual‐task assessment in the NHPT, participants were given a word‐generation task. They were asked to generate as many words as possible, beginning with the letter “K” for their dominant extremity and with the letter “S” for their nondominant extremity throughout the test. The completion time and number of correct words were recorded.

During the word‐generation tasks, we attempted to minimize the learning effect by using three different letters for three different tasks. The three letters (K, A, and S) are the first three letters used in the word list generation tests in neuropsychological batteries adapted to Turkish (Öktem, 1994).

The time difference between the single and dual tasks was calculated, and the percentage was taken. The motor dual‐task cost was calculated for both 30s WT and NHPT using the formula: (single‐task performance—dual‐task performance)/(single‐task performance) × 100 (Baddeley et al., 1997). Additionally, we reported absolute dual‐task performance for 30s WT and NHPT to reflect actual walking and upper extremity function with secondary cognitive load. While dual‐task cost is a widely accepted metric for evaluating the impact of multitasking on performance, we also incorporated absolute measures of dual‐task performance to provide a comprehensive understanding of the actual performance levels achieved when participants engage in both tasks simultaneously. This approach is supported by previous literature and is particularly relevant to our research, which aims to examine daily life challenges and actual performance levels of people with MS (Hillel et al., 2019; Shema‐Shiratzky et al., 2020).

#### 2.2.4 Self‐reported work difficulties

The Multiple Sclerosis Work Difficulties Questionnaire‐23 (MSWDQ‐23) is a scale consisting of 23 items divided into three subscales: 11 items for psychological and cognitive barriers, 8 items for physical barriers, and 4 items for external barriers (Honan et al., 2014). Participants are asked to rate the frequency of each difficulty they have experienced because of their MS disease in the past four weeks on a 5‐point Likert‐type scale. Each item on the scale is scored between 0 (never) and 4 (almost always). Each subscale score is calculated as a percentage. Total MSWDQ‐23 scores are calculated by averaging the scores of the three subscales. High scores indicate high perceived work difficulties. The Turkish validity and reliability of the MSWDQ‐23 have been demonstrated (Kahraman et al., 2019). The MSWDQ‐23 was only administered to people with MS who were employed.

#### 2.2.5 Cognitive function

The Brief International Cognitive Assessment in Multiple Sclerosis (BICAMS) is a widely used method for evaluating cognitive functions in people with MS (Langdon et al., 2012). It was specifically designed for assessing cognitive abilities and does not require extensive time, or specialized training, or equipment for evaluation (Langdon et al., 2012). The BICAMS comprises three tests: the SDMT, the California Verbal Learning Test (CVLT), and the Brief Visuospatial Memory Test‐Revised (BVMT‐R). These tests measure working memory, verbal memory, and visual memory, respectively (Langdon et al., 2012). The BICAMS has been adapted and validated for use in the Turkish population (Ozakbas et al., 2017).

### 2.3 Statistical analysis

The IBM SPSS Statistics software (Version 29.0 Armonk) was used for data analysis. The normal distribution of the data was ensured by examining the Shapiro–Wilk test results and histograms. Descriptive statistics were presented as mean (standard deviation) for normally distributed variables and median (interquartile range) for non‐normally distributed variables. Categorical variables were presented as percentages.

To compare the two groups, chi‐square test was used for categorical variables, Mann—Whitney U test was used for non‐normally distributed continuous variables, and independent *t*‐test was used for normally distributed continuous variables. Binary logistic regression analysis was conducted to investigate the predictors of employment status using the variables that showed significant differences between the employed and unemployed participants. As both groups had significant differences in sex and education level, the analyses were adjusted for these variables. Cox and Snell's *R* ^2^ and Nagelkerke's *R* ^2^ were used as measures of goodness‐of‐fit for binary logistic regression models. Correlations in the employed group were investigated using Spearman correlation analysis and correlation coefficients between .1 and .29 were interpreted as weak, between .3 and .49 as moderate, and between .5 and 1.0 as strong.

## 3 RESULTS

The study involved 84 people with MS, half of whom were unemployed. Females had higher unemployment rates (*p* = .007), and employed participants had higher education levels compared to the unemployed participants (*p* < .001). Results are presented in Table 1.

**TABLE 1:** Comparison of the basic demographic and clinical characteristics of the participants.

	Employed (n = 42)	Unemployed (n = 42)	*p*‐Value
Age, years [mean (SD)]	38.71 (1.25)	41.33 (1.23)	.147
Body mass index, kg/m^2^ [mean (SD)]	25.09 (3.98)	25.79 (5.27)	.498
Sex, female [n (%)]			
Female	30 (71.4)	40 (95.2)	.007*
Male	12 (28.6)	2 (4.8)	
Education [n (%)]			
Primary school, 0–5 years	0	12 (28.6)	<.001*
Secondary school, 6–8 years	3 (7.1)	9 (21.4)	
High school, 9–12 years	8 (19)	16 (38.1)	
University, >12 years	31 (73.8)	15 (11.9)	
EDSS, 0–10 [median (IQR)]	1.75 (1.37–2.5)	2.0 (1.5–3.62)	.08
Disease duration, years [median (IQR)]	13.0 (6.87–17.25)	11.0 (8–15.75)	.468
Unemployment duration, years [median (IQR)]	N/A	4 (2–12.5)	N/A
Employment duration, years [median (IQR)]	14.75 (10–23)	5 (2–10)^a^	N/A

*p < .05.

^a^ Represents the duration of employment before unemployment.

EDSS, Expanded Disability Status Scale; IQR, interquartile range; SD, standard deviation.

Employed people with MS had better scores in T25FW, SDMT, CVLT, and BVMT‐R compared to unemployed counterparts (*p* < .05). Employed people with MS had better dual‐task performance, as evidenced by higher scores in absolute dual‐task 30s WT distance and correct answers, and dual‐task NHPT correct answers (*p* < .05). Moreover, employed people with MS had fewer self‐reported dual‐task difficulties, presented by significantly lower scores on the DIDA‐Q subscales (*p* < .05). However, there were no significant differences in 30s WT and NHPT dual‐task cost scores, or NHPT between the employed and unemployed groups (*p* > .05). Table 2 presents the cross‐sectional comparison of walking, cognitive function, single and dual‐task performance between employed and unemployed people with MS.

**TABLE 2:** Cross‐sectional comparison of walking, cognitive function, single and dual‐task performance between employed and unemployed people with MS.

	Employed (n = 42)	Unemployed (n = 42)	*p*‐Value
T25FW, s	4.57 (4.15–5.22)	5.57 (4.78–7.1)	<.001*
SDMT	54.51 (10.39)	43.12 (12.88)	<.001*
CVLT	61.12 (11.91)	52.19 (14.34)	.003*
BVMT‐R	29.05 (4.64)	23.9 (5.97)	<.001*
30sWT, m	44.48 (7.74)	39.33 (7.92)	.003*
Absolute dual‐task 30s WT, m	40 (34–43)	35 (40.31–40)	.002*
Absolute dual‐task 30s WT, correct answers	7.81 (2.95)	4.57 (2.22)	<.001*
30sWT motor dual‐task cost, %	11.73 (6.82–16.67)	10.75 (11.89–14.29)	.385
NHPT, s	20.09 (17.7–22.45)	21.44 (20.73–26.16)	.105
Dual‐task NHPT, s	24.3 (20.8–33.4)	27.94 (24.47–32.6)	.149
Dual‐task NHPT, correct answers	7.73 (2.59)	5.33 (2.74)	<.001*
NHPT, motor dual‐task cost, %	−15.62 (−43.63 to −8.23)	−19.2 (−15.58 to −8.85)	.996
DIDA‐Q, Upper Extremity	2.5 (0–7)	6 (2.2–10)	.006*
DIDA‐Q, Balance‐Mobility	2 (0–7)	5.5 (2.2–10)	.020*
DIDA‐Q, Cognition	1.5 (0–3)	5 (1.1–10)	<.001*

*p < .05.

Variables are presented as mean (SD) or median (IQR).

30sWT, 30‐s Walk Test; DIDA‐Q, Dual‐task Impact on Daily‐living Activities Questionnaire; NHPT, Nine‐Hole Peg Test.

The results presented in Table 3 show the findings of the binary logistic regression analysis aimed to predict employment status based on various factors. The analysis was adjusted for sex, education level, and EDSS. The results indicate that lower scores in absolute dual‐task 30s WT correct answers (OR = 1.81, *p* < .001) and NHPT (OR = 1.44, *p* = .019) correct answers, and higher scores in DIDA‐Q cognition subscale (OR = 1.20, *p* = .037) were significantly associated with higher odds of being unemployed. Absolute dual‐task 30s WT and NHPT correct answers accounted for 70 and 63% of the total variance, respectively, whereas the DIDA‐Q cognition subscale accounted for 61%. On the other hand, the other variables were not significantly associated with employment status. The Cox and Snell *R* ^2^ values range from .428 to .527, while the Nagelkerke's *R* ^2^ values range from .571 to .703, suggesting that these models provide an adequate fit for the data.

**TABLE 3:** Binary logistic regression analysis results to predict employment status.

	OR	95%CI (Lower—Upper)	*p*‐Value	Cox and Snell *R* ^2^	Nagelkerke's *R* ^2^
T25FW, s	1.27^a^	.74–2.17	.382	.433	.578
SDMT	1.06^b^	.99–1.13	.085	.488	.598
CVLT	1.02^b^	.97–1.06	.493	.428	.571
BVMT‐R	1.04^b^	.93–1.17	.501	.428	.571
30sWT, m	1.06^b^	.95–1.17	.306	.435	.580
Absolute dual‐task 30sWT, m	1.04^b^	.93–1.15	.499	.431	.575
Absolute dual‐task 30sWT, correct answers	1.81^b^	1.27–2.57	<.001*	.527	.703
30sWT dual‐task cost, %	1.04^a^	.94–1.17	.400	.433	.577
NHPT, s	1.08^a^	.97–1.21	.179	.442	.590
Absolute dual‐task NHPT, s	1.02^a^	.96–1.07	.557	.430	.574
Absolute dual‐task NHPT, correct answers	1.44^b^	1.06–1.94	.019*	.472	.629
NHPT, dual‐task cost, %	1.00^a^	.99–1.02	.754	.429	.572
DIDA‐Q, Upper Extremity	1.07^a^	.92–.124	.374	.433	.578
DIDA‐Q, Balance‐Mobility	1.01^a^	.86–1.20	.867	.428	.571
DIDA‐Q, Cognition	1.20^a^	1.01–1.43	.037*	.460	.614

*p < .05, adjusted for sex, education level, and EDSS.

^a^ Reference: unemployed.

^b^ Reference: employed.

30s WT, 30‐s Walk Test; CI, confidence interval; DIDA‐Q, Dual‐task Impact on Daily‐living Activities Questionnaire; EDSS, Expanded Disability Status Scale; NHPT, Nine‐Hole Peg Test; OR, odds ration.

**FIGURE 1:**
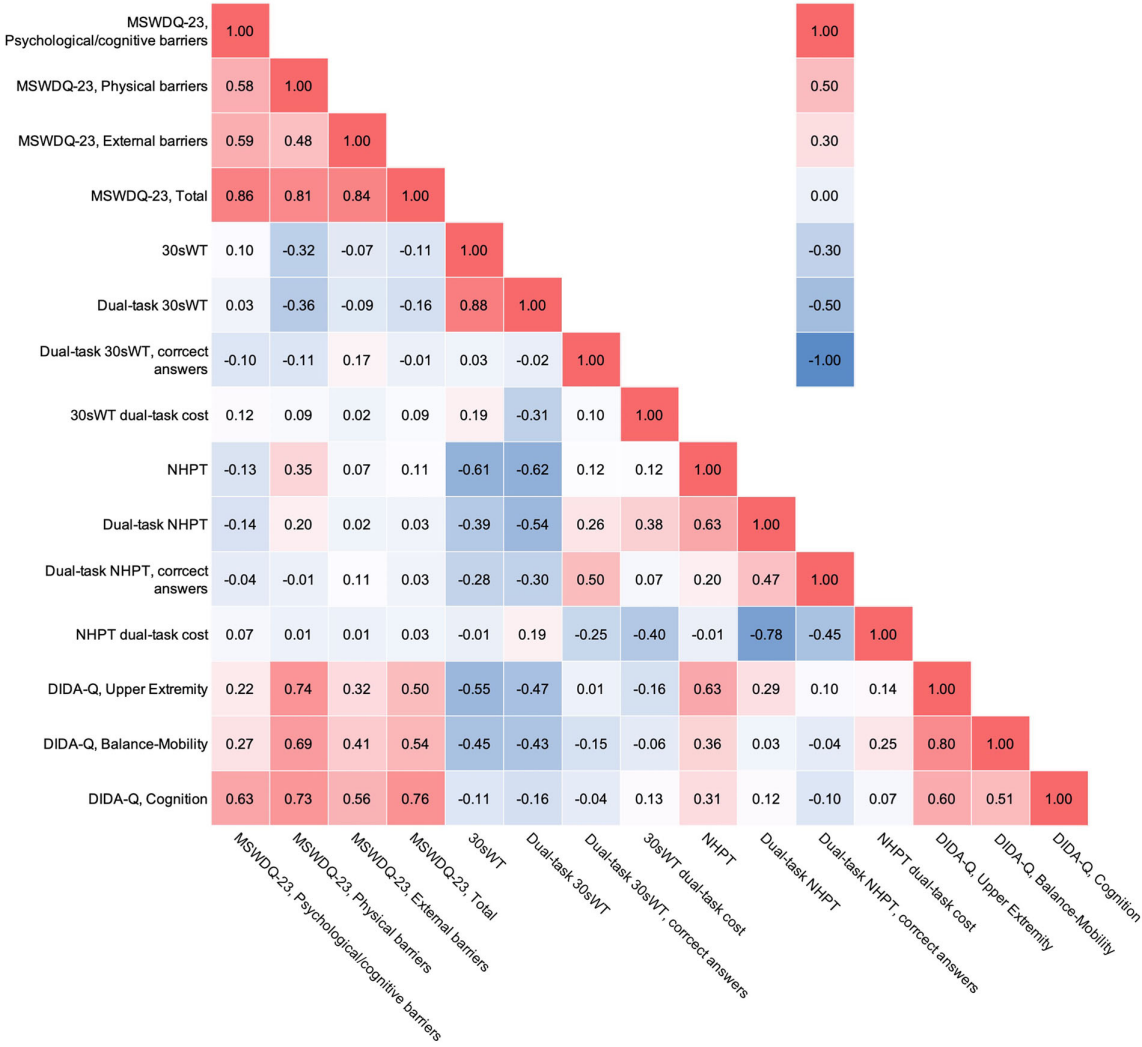
Correlation coefficients between work difficulties and other variables adjusted for sex and education level in employed people with MS (n = 42).

In Figure 1, correlation coefficients between the MSWSDQ‐23 and other study variables in employed people with MS were presented. Among employed people with MS, the DIDA‐Q subscales showed moderate to strong correlations with all subscales and total scores of MSWSDQ‐23. The other investigated variables showed weak to moderate correlations with subscale and total scores of MSWSDQ‐23.

## 4 DISCUSSION

This study showed that employed people with MS had better dual‐task performance and reported fewer dual‐task difficulties. Lower numbers of correct answers in a dual‐task walking test (30s WT) and manual dexterity test (NHPT) and higher dual‐task cognitive impact on activities of daily living (DIDA‐Q cognition subscale) were significantly associated with higher odds of being unemployed. Higher dual‐task cognitive impacts on activities of daily living were strongly associated with higher work difficulties among employed people with MS.

Previous studies have established that employment for people with MS is influenced by various factors, encompassing both motor and cognitive aspects. These factors contribute to the individual's employment status and the challenges they encounter in the workplace (Benedict et al., 2005; Julian et al., 2008; Morrow et al., 2010; O'Connor et al., 2005; Rodriguez Llorian et al., 2022; Simmons et al., 2010; Smith & Arnett, 2005; Strober et al., 2012). In our study, we showed that cognitive performance during cognitive‐motor dual‐task activities as a potential predictor of employment in people with MS. This finding is not unexpected, as dual‐task performance serves as a significant indicator of everyday functioning (Shema‐Shiratzky et al., 2020). Given that most daily activities involve the execution of dual tasks, it has been recognized as a comprehensive and ecologically valid measure (Pedullà et al., 2020).

Work life predominantly involves situations that demand divided attention, characterized by the simultaneous execution of motor and cognitive tasks rather than isolated motor or cognitive activities. Therefore, the ability to maintain performance in these dual‐task situations without compromising productivity may enhance individuals' self‐confidence, social participation, and overall work engagement. Our logistic regression analysis provides support for the notion that assessing motor and cognitive functions separately, employing measures such as the walking (T25FW), upper extremity function (NHPT), or cognitive function (SDMT), does not adequately predict employment status. However, it is the cognitive performance during dual‐task activities, rather than motor performance, that significantly influences employment outcomes.

The level of disability plays a significant role in determining employment outcomes for people with MS. Even at the early stages of disability, a study found an unemployment rate of 25% and observed a loss in productivity (Rodriguez Llorian et al., 2022). Therefore, it is crucial to identify factors that impact employment, particularly those that can be modified, as they have a substantial influence on the most productive period of a person's life. In our study, we observed that both groups of participants had relatively lower levels of disability. Learmonth et al. (2017) found that mildly disabled people with MS exhibited minimal and nonsignificant impairment in dual‐task walking performance when engaged in a second cognitive task. The association between cognitive performance, but not motor performance during dual‐task activities, with employment outcomes is likely attributable to the low disability level and preserved motor function, including hand function and gait, among the participants in our study.

Our study found that the impact of information processing speed, assessed by the SDMT, was not linked to unemployment. Nevertheless, cognitive performance during dual tasks was found to increase the risk of unemployment, which supports our hypothesis. Previous studies have also highlighted the significant effect of SDMT on employment status (Benedict et al., 2005; Morrow et al., 2010; Rodriguez Llorian et al., 2022; Strober et al., 2012). However, Smith and Arnett's (2005) study contradicted this finding by demonstrating that comprehensive assessments of cognitive dysfunction, including various tests such as the Controlled Oral Word Association test, 7/24 Spatial Recall test, verbal selective reminding test, Paced Auditory Serial Addition Test, Tower of Hanoi test, and SDMT, were not associated with employment status. It is possible that our study participants, who exhibited mild disability, had not yet reached the threshold at which SDMT would impact their work. Conversely, Smith and Arnett's study included a larger proportion of more disabled people with MS compared to our study (Smith & Arnett, 2005). Therefore, considering the inconclusive results in the existing literature, we propose that dual‐task difficulties may better predict employment status in people with MS, particularly those with mild disability. However, it is important to note that further confirmation of our speculation would require a longitudinal study.

In recent years, patient‐reported outcomes have garnered considerable interest due to their potential utility in capturing the nuances of daily life challenges that are challenging to assess solely through laboratory‐based testing. Alongside the well‐documented impact of MS on unemployment rates, people with MS have reported encountering work‐related challenges and a decline in productivity (Conradsson et al., 2020; Ernstsson et al., 2016; Rodriguez Llorian et al., 2022; Vitturi et al., 2022). In our study, we examined the perceived dual‐task difficulties within the cognitive domain, encompassing activities such as walking while remembering, engaging in conversations, listening, and maintaining attention to traffic. Notably, we found a substantial association between the perceived dual‐task difficulties experienced by the employed participants and their reported work‐related challenges. Furthermore, although there was no difference in dual‐task cost between the groups, it can be argued that the higher scores on all subtests of the DIDA‐Q in the unemployed group reflect the difficulties that the items of the DIDA‐Q represent in the occupational context, such as walking and talking, walking, and performing mental arithmetic tasks, walking calling someone. These discoveries underscore the significance of employing self‐reported measurement tools and suggest that dual‐task difficulties may serve as an indicator of the difficulties individuals face in balancing their work and personal lives.

In employed people with MS, the DIDA‐Q demonstrated moderate to strong correlations with all MSWSDQ‐23 subscales and the overall scores. Conversely, the remaining examined variables displayed relatively weak to moderate correlations with both the subscales and total scores of the MSWSDQ‐23. Researchers and clinicians can consider using the DIDA‐Q as a valuable tool for evaluating and monitoring dual‐task difficulties related to work difficulties in this population. While other investigated variables may not be as strongly correlated, they still provide some insight into the factors that influence work difficulties in people with MS. Moreover, in practical terms, frequently administering tests as dual‐tasks instead of individually can yield valuable insights into the work environment.

In our study, there was a significant difference in sex and education levels between the employed and unemployed people with MS. Therefore, we adjusted for sex and education levels in our regression analysis to discern the specific influence of dual‐task performance on employment status. However, it is essential to recognize the intertwined nature of education, employment, and dual‐task performance, and their collective impact on the study's findings.

There is a positive relationship between cognition, education, and employment in people with MS that greater education results in better baseline processing speed and is protective with respect to employment status (Conway et al., 2022). Conversely, people with lower education levels can face distinct challenges, experiencing comparatively lower dual‐task performance scores and a higher prevalence of unemployment. These disparities may be attributed to a variety of factors, including limited access to quality education, socioeconomic constraints, and potentially reduced cognitive reserves. However, according to the best of our knowledge, no study has investigated the relationship between education level and dual‐task performance.

While our regression analysis accounted for education as a covariate, our observations suggest that the impact of education can extend beyond its role as a confounding factor. In light of these findings, it is evident that future research should delve deeper into the mechanisms through which education level influences the dual‐task performance and employment status.

Our study presents original insights into the determinants of employment and work challenges in people with MS; nevertheless, certain limitations must be taken into account. First, our sample predominantly consists of people with MS and mild disability. This characteristic can be viewed as advantageous, as it accurately represents the prevailing range of MS cases, but it also poses a limitation by potentially impeding the generalizability of our findings to people with MS with moderate to severe disability. Moreover, owing to the cross‐sectional nature of our study design, we are unable to capture the dynamic fluctuations in employment status and work difficulties among people with MS experiencing dual‐task impairment. Since we did not evaluate the single cognitive task performance, we were unable to calculate the cognitive dual‐task cost. However, it is important to highlight that, when taking this limitation into account, the reliability of the cognitive dual‐task cost is low in the MS population (Veldkamp et al., 2019). In people with MS, impaired dual‐task performance has been identified as a potential factor contributing to unemployment. However, the specific relationship between unemployment and dual‐task performance has not been explored in the present study. Consequently, it remains unclear whether unemployment itself influences dual‐task performance in people with MS. Further investigation is warranted to shed light on the potential impact of unemployment on dual‐task performance. Last, in our study, we chose to use the BICAMS assessment, which primarily focuses on memory‐related cognitive functions. This decision was influenced by the practicality of the BICAMS, which requires only 15 min to administer and covers the most affected cognitive domains in people with MS. However, we acknowledge that a more comprehensive assessment encompassing a broader range of cognitive domains would provide a more complete picture of relationship between cognitive function, dual‐task performance, and employment in people with MS.

In conclusion, the current study reveals associations between cognitive performance during cognitive‐motor dual‐task assessments, self‐reported difficulties in dual‐task situations involving cognitive activities, and unemployment in people with MS. These associations suggest that these factors may play a role in employment status among people with MS. Additionally, our findings highlight a strong relationship between self‐reported dual‐task difficulties and workplace challenges among employed people with MS. While our study provides valuable insights into these associations, it is important to acknowledge the cross‐sectional nature of our design, which limits our ability to establish causality. Therefore, rather than making definitive predictions, our results suggest that further research is needed to explore the potential impact of interventions aimed at improving dual‐task performance on employment outcomes for people with MS.

## AUTHOR CONTRIBUTIONS

**Turhan Kahraman**: Conceptualization; methodology; writing—original draft; writing—review and editing; formal analysis; project administration; supervision; visualization. **Hasretgul Temiz**: Conceptualization; investigation; writing—original draft; methodology; project administration; formal analysis. **Zuhal Abasiyanik**: Conceptualization; investigation; writing— original draft; formal analysis; project administration; methodology. **Cavid Baba**: Methodology; investigation; resources. **Serkan Ozakbas**: Conceptualization; methodology; writing—review and editing; resources; investigation.

## CONFLICT OF INTEREST STATEMENT

Authors declare there is no conflict of interest.

### PEER REVIEW

The peer review history for this article is available at https://publons.com/publon/10.1002/brb3.3299

## Data Availability

The data that support the findings of this study are available on request from the corresponding author. The data are not publicly available due to privacy or ethical restrictions.
